# The quest for environmental analytical microbiology: absolute quantitative microbiome using cellular internal standards

**DOI:** 10.1186/s40168-024-02009-2

**Published:** 2025-01-27

**Authors:** Chunxiao Wang, Yu Yang, Xiaoqing Xu, Dou Wang, Xianghui Shi, Lei Liu, Yu Deng, Liguan Li, Tong Zhang

**Affiliations:** 1 Environmental Microbiome Engineering and Biotechnology Laboratory, Center for Environmental Engineering Research, Department of Civil Engineering, The University of Hong Kong, Pok Fu Lam, Hong Kong, China; 2 School of Public Health, The University of Hong Kong, Hong Kong, China; 3 Macau Institute for Applied Research in Medicine and Health, Macau University of Science and Technology, Macau, China; 4 State Key Laboratory of Marine Pollution, City University of Hong Kong, Hong Kong, China; 5 Division of Applied Oral Sciences & Community Dental Care, Faculty of Dentistry, The University of Hong Kong, Hong Kong, China; 6 Department of Science and Environmental Studies, The Education University of Hong Kong, 10 Lo Ping Road, Tai Po, New Territories, Hong Kong, China

**Keywords:** Absolute quantification, Microbiome, Environmental analytical microbiology, Cellular internal standard, Standardization

## Abstract

**Background:**

High-throughput sequencing has revolutionized environmental microbiome research, providing both quantitative and qualitative insights into nucleic acid targets in the environment. The resulting microbial composition (community structure) data are essential for environmental analytical microbiology, enabling characterization of community dynamics and assessing microbial pollutants for the development of intervention strategies. However, the relative abundances derived from sequencing impede comparisons across samples and studies.

**Results:**

This review systematically summarizes various absolute quantification (AQ) methods and their applications to obtain the absolute abundance of microbial cells and genetic elements. By critically comparing the strengths and limitations of AQ methods, we advocate the use of cellular internal standard-based high-throughput sequencing as an appropriate AQ approach for studying environmental microbiome originated from samples of complex matrices and high heterogeneity. To minimize ambiguity and facilitate cross-study comparisons, we outline essential reporting elements for technical considerations, and provide a checklist as a reference for environmental microbiome research.

**Conclusions:**

In summary, we propose absolute microbiome quantification using cellular internal standards for environmental analytical microbiology, and we anticipate that this approach will greatly benefit future studies.

Video Abstract

**Graphical Abstract:**

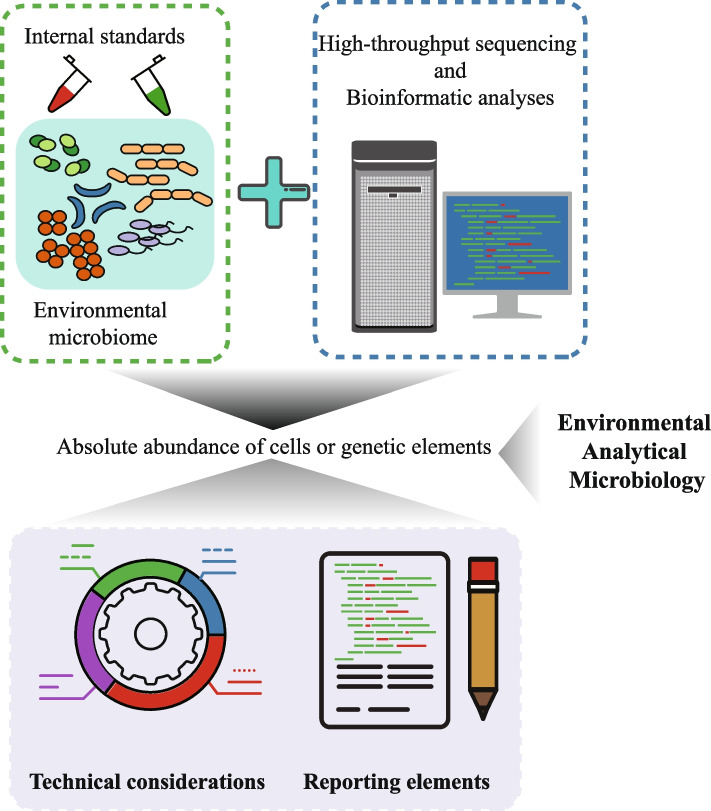

**Supplementary Information:**

The online version contains supplementary material available at 10.1186/s40168-024-02009-2.

## 1. Introduction

**Fig. 1:**
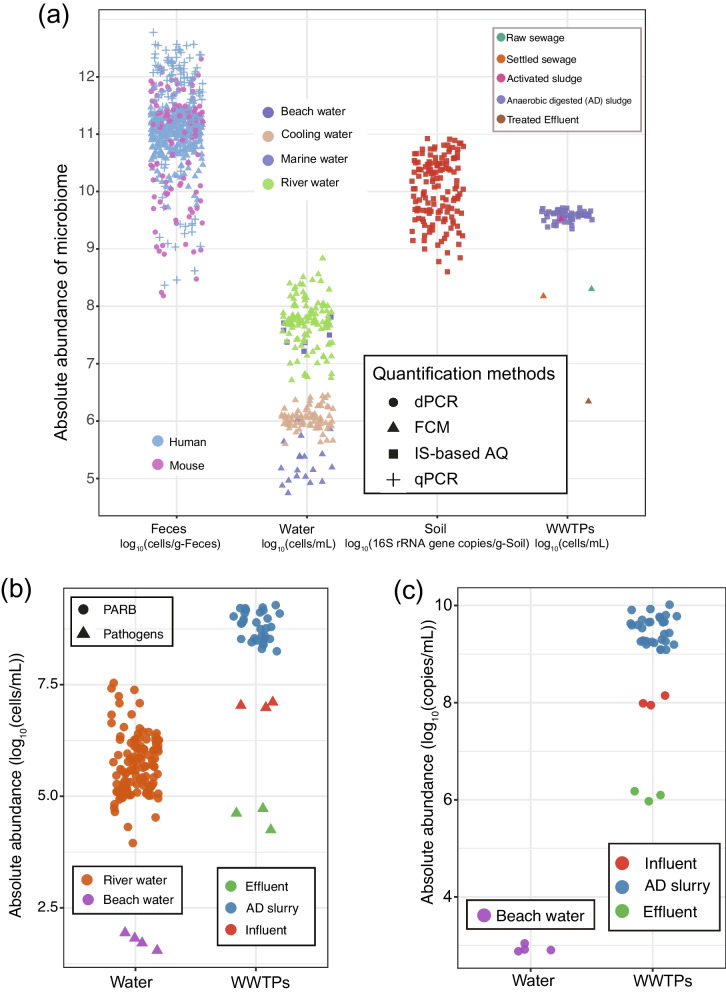
Absolute abundance of environmental microbiome, including total microbial loads across different habitats using diverse AQ methods (**a**) (data source: [6, 18–26]); pathogens and pathogenic antibiotic-resistant bacteria (PARB) density in water samples and wastewater treatment works (WWTPs) (**b**) (data source: [1, 23, 27]); and ARG concentrations in water samples and WWTPs using IS-based AQ methods (**c**) (data source: [1, 6, 27])

The environmental microbiome, a complex assembly of microbial cells and their genetic constituents, plays multifaceted roles in natural biochemical processes and engineered systems [1–5], and has advantageous or deleterious impacts on humans, animals, plants, and the environment [6]. Analogous to environmental analytical chemistry, which investigates the distribution and concentrations of chemical pollutants [7], we propose a new discipline “Environmental Analytical Microbiology (EAM),” which treats microbes and related genetic elements in the environment as analytes. This will encompass the documentation of various microbial cells in different habitats (Fig. 1a), and enable the spatiotemporal monitoring of microbial pollutants such as pathogens (Fig. 1b) [1, 6] and microbial genetic elements like antibiotic resistance genes (ARGs) (Fig. 1c) [8, 9]. Additionally, EAM facilitates the profiling of the physiological properties, such as cell growth, metabolic pathways, and activity, of desired functional microbes and their response to changes in environmental variables [10, 11]. By integrating EAM with appropriate management practices, it is possible to augment the favorable effects of the microbiome on humans, animals, plants, and the environment. This integration involves establishing connections between microbiomes and the physio-chemical conditions of systems [12], pinpointing biomarkers to assess the performance of engineered systems [13, 14], mitigating the negative impacts through bioaugmentation remediation technology [15], and enhancing the beneficial effects on the environment through enriching functional populations to accelerate rates of biochemical reactions in element cycling, such as nitrogen fixation or the mineralization of organic matter [16, 17].

The advent of high-throughput sequencing technologies has revolutionized the way researchers explore the microbial world and profoundly influenced the field of genomics, offering numerous benefits such as larger sample sizes (such as global and regional surveillance), culture-independent analysis, reduced labor intensity, comprehensive scanning, in-depth investigations, and rapid detection [1, 28, 29]. The incorporation of this rapidly advancing technology into environmental studies has the potential to greatly increase the efficiency of the analytical processes and yield a more comprehensive understanding of the examined microbiome. However, a few challenges reduce the reliability of quantitative results generated from high-throughput sequencing data [30]. First, technical bias can be introduced at any stage of microbiome analysis, from sample collection to results interpretation [31, 32]. Variability can easily arise from technical factors such as sampling strategy (e.g., grab vs. composite sampling [33]), sample preservation and storage (e.g., the effect of ethanol concentration [32, 34], storage temperature, and duration [35]), DNA extraction methods and/or kits [34, 36, 37], biological replication [38], technical replication [39, 40], sequencing library preparation [41, 42], uneven sequencing of samples in a multiplexed run [28], and different sequencing platforms [43].

To eliminate potential biases caused by these sources of variability and conduct EAM, microbiome quantification should be performed in absolute values for inter-sample comparison and reliable statistical analyses. It helps rectify for possible compositional artifacts [44] associated with relative abundances [18, 31]. Without considering variations in total microbial loads (also referred as cell density, i.e., cell counts per unit mass/volume), the compositional nature of the relative data obtained from the sequencing of different samples can result in misinterpretations of microbial findings [18, 19, 45, 46]. Since the compositional data is constrained to a constant sum, an increase in one taxon’s abundance inevitably leads to a concurrent decrease in the abundance of other taxa [19, 20]. This characteristic can lead to high false-positive rates in differential abundance analyses [47–49], introduce spurious correlations [30], be especially severe when communities have dominant taxa [50], and miss possible correlation pairs [18]. All of these factors hinder inter-sample and inter-study comparisons. Additionally, biological and ecological insights, including microbiota-host interplay [18] and inter-species interactions engineered systems [21], can be enhanced by the knowledge of absolute abundances [45].

To achieve absolute quantification (AQ), various methods can be adopted to determine the absolute abundance of microbial taxa via known “anchor” points that convert relative data into absolute values [19, 45]. In this review, we categorize the current methods into two groups: (i) incorporating relative abundance with total microbial load and (ii) internal standard (IS)-based AQ (also known as IS-facilitated AQ). We argue that conventional cultivation-based and direct counting methods, while backed by well-established protocols, may not be able to fulfill the key criteria of applicability, reliability, rapidity, and affordability, particularly for complicated environmental samples, owing to their inherent limitations. These conventional methods often face challenges with the complexity and variability of these samples, including diverse matrix effects, leading to less reliable results, longer processing times, and increased costs. Instead, we propose that IS-based metagenomic quantification can benefit EAM because it is applicable for (i) diverse environmental samples, regardless of whether cells are in a free-living state or in flocs; (ii) independent of cultivation, as the majority of bacteria in natural or engineered systems have not been isolated [51]; and (iii) wide-spectrum scanning, including the enumeration of both single species and higher phylogenetic taxa (e.g., genera or classes, phyla). However, this approach has several drawbacks, including potential biases arising from the selection of IS and sequencing technologies, the requirement for specialized computational resources and expertise to analyze the data and interpret quantitative results, and relatively high limits of detection (LoD) compared with the conventional methods. Furthermore, we outline key elements for technical consideration, aimed at improving the reliability and feasibility of AQ methods, and provide an element checklist as a reference for future studies in the field of the environmental microbiome to advance EAM.

### 2. Estimation of total microbial load

**Fig. 2:**
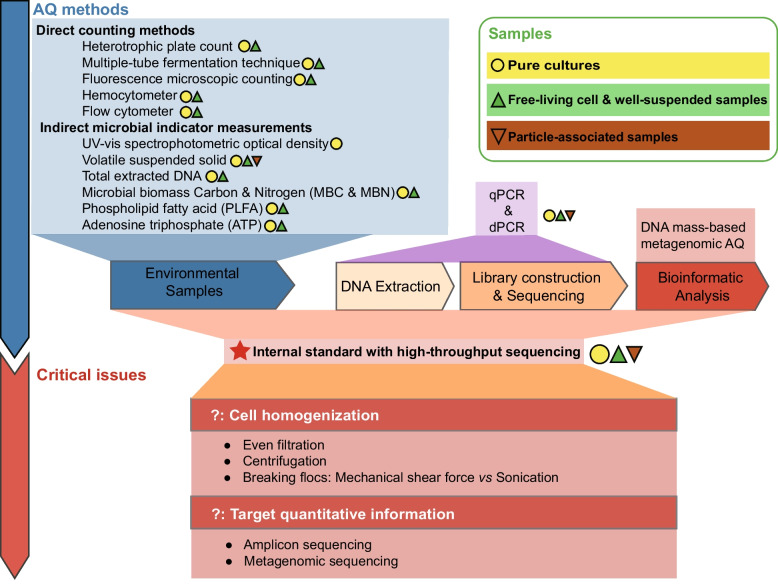
Absolute quantification (AQ) methods and critical issues to be addressed

The total microbial load can be estimated using various methods of three groups: (1) direct counting including cultivation, microscopic counting, and flow cytometry (FCM); (2) indirect microbial indicator measurements, including biomass dry weight, total extracted DNA, OD_600_, microbial biomass carbon and nitrogen (MBC and MBN), phospholipid fatty acid (PFLA), and adenosine triphosphate (ATP); and (3) molecular methods including quantitative polymerase chain reaction (qPCR) and digital PCR (dPCR), and computation-based metagenomic absolute quantification. Each method has advantages and shortcomings and is affected by diverse factors (as summarized in Table S1 and Fig. 2).

#### 2.1 Direct counting methods

The heterotrophic plate count (HPC), measured in colony-forming unit (CFU) [52], and multiple-tube fermentation technique, measured in most probable number (MPN) [53], are cultivation-based methods for counting cell numbers in water and wastewater samples [54]. The major limitation is the underestimation of the total number of cells because the “unseen majority” is non-culturable at given conditions [55]. To include cells in a viable but nonculturable state, dead and non-culturable cells, microscopic counting via fluorescence microscopes, and hemocytometers can be employed. The fluorescence microscopic method involves staining cells with DNA-specific dyes or probes, capturing cells on the membrane, and enumerating them under a microscope [51, 56–59]. Additionally, the hemocytometer, a specialized counting chamber, allows cells suspended in a sample to be counted under a light microscope [60]. However, these above methods are largely affected by the cell distribution status of the samples and the skills of the operators. Typically, pre-treatments or specialized devices (such as a multi-volume hemocytometer) are required to ensure the number of cells within measurable ranges [60, 61].

FCM has gained considerable attraction for cell number enumeration because of its highly informative nature in counting various cells in microbial communities [62, 63], ability to distinguish live and dead cells using different DNA dyes [64], and advantages of high accuracy, reproducibility, rapid processing, automation potential, and cost-effectiveness [55]. For example, FCM can deliver reproducible results within 15 min with relative standard deviations less than 3% [55]. However, FCM faces challenges such as potential bias during sample preparation [55], a lack of a universal FCM setting and analytical protocol [21], and interference from cell debris and aggregates [22, 51]. FCM counting is more suitable for environmental samples with low biomass and well-dispersed cells, such as drinking water [62], cooling water samples [46], and river samples [23].

As an alternative to DNA dyes, oligonucleotide probes targeting ribosomal RNA (rRNA) genes can be used for cell identification via fluorescence in situ hybridization (FISH); when it combined with FCM and confocal laser scanning microscopy, free-living and aggregated microbes can be quantified in absolute values [51, 65, 66]. Catalyzed reporter deposition FISH (CARD-FISH) can amplify signals from low abundance or activity microbes, recovering an average of 94% of cells [67], and performs well in monitoring and localizing microbial populations within particles [68]. However, the application of FISH-facilitated methods for absolute quantification in complex samples is limited due to high demand on operating experience, sample preparation challenges, and target molecule accessibility [69, 70].

#### 2.2 Indirect microbial indicator measurements

A variety of indirect microbial indicators can be used for approximating cell numbers, and these methods necessitate the linkage between actual cell counts and the parameters used in a specific method. The volatile suspended solid (VSS) and total DNA amount (in the unit of µg DNA per mg sample) serve as proxies for biomass in wastewater treatment systems [71–73], and the microbial load [74], respectively. These estimations may be imprecise because non-microbial organic particles in VSS interfere with measurements, and non-bacterial DNA sources and variations in microbial genome size affect the conversion from total DNA to cell numbers [74]. The UV–Vis spectrophotometric optical density (OD) at a wavelength of 600 nm is a commonly employed method to determine the number of pure culture microorganisms in liquid culture [75, 76], but is not commonly used for environmental samples. Other methods involve the extraction of relevant materials from cells to estimate the microbial cell numbers, including (i) MBC [77–79] and MBN [80]; (ii) PLFA with an empirical value of 1.40 × 10^−8^ nmol bacterial PLFA/cell [81]; and (iii) ATP with an empirical value of 1.75 × 10^−10^ nmol ATP/cell [73, 77, 82] and other case-specific conversation values [83]. However, the calibration between cell numbers and values of MBC, MBN, PLFA, and ATP is needed, making it very challenging for environmental samples containing diverse microbes and cells with different activities.

#### 2.3 Molecular methods

qPCR and dPCR, both reliant on PCR technology and fluorescent signals, provide quantitative data on microbial nucleic acid targets in diverse environments [40, 84]. The advantages of these methods include high analytical sensitivity and specificity, high throughput for multiple genes and samples, and extensive dynamic ranges, and rapid turnaround times [40]. qPCR achieves quantification by comparing Ct values with a standard curve generated from DNA fragment with known copy numbers, while dPCR partitions PCR reactions, measures fluorescence signals, and applies Poisson statistics for quantification [40, 85]. dPCR offers several advantages over qPCR for quantification, including enhanced sensitivity, greater tolerance to PCR inhibitors, and not relying on a standard curve [86, 87]. However, qPCR is generally more cost-effective and faster, and has well-established protocols [88, 89]. One critical part of PCR is the need for specific primer pairs, which may not be easily accessible depending on the study objectives, necessitating design and evaluation before application [90]. Importantly, contamination is a common issue [40], particularly for samples with low total microbial loads [19]. Moreover, the main challenge for qPCR and dPCR is amplification bias and the matrix effect of complex environmental samples, which can potentially obscure the real quantitative results [91–94].

Like qPCR and dPCR quantification methods, the DNA mass-based metagenomic absolute quantification method assumes a 100% extraction efficiency which is consistent across all studied samples [2]. The DNA mass-based metagenomic absolute quantification method involves coverage (*D*) of microbial cells or genetic elements in the sequencing reads (Eq. 1 [95]) (Table 1), and the sequencing rate of DNA (*S*_*r*_), which is the ratio between the DNA mass of metagenomic sequenced reads (*m*) and the total extracted DNA mass (*M*) (calculated using Eqs. 2 and 3) [2]. The absolute abundance can be calculated by dividing the coverage by the sequencing rate [2]. In the reality, the quantification of total extracted DNA mass (*M*) might be influenced by instrumental measurement, while bioinformatic analysis, including the selection of alignment algorithms, tools, and cutoffs might affect coverage (*D*) values.

**Table 1:** Equations used in AQ methods

Eq. No	Equations	[Ref.]
1	$D=\frac{{\sum }_{i=1}^{n}{R}_{i }}{L}$	[95]
2	$m=\frac{{[(A}_{n}\times 313)+{(T}_{n}\times 304){+(C}_{n}\times 289)+{(G}_{n}\times 329)]\times 2}{6.02\times {10}^{23}}\times {10}^{-9}$	[2]
3	${S}_{r} =\frac{m}{M}=\frac{m}{C\times V}$	[2]
4	$SF=\frac{N}{{D}_{IS}}$	[1, 21]
5	$X=N(\frac{1}{A}-1)$	[31]
6	$X=\frac{Q}{\eta }$	[96]
7	$\eta (\%)=\frac{{N}_{D}}{N}$	[96]
8	$\eta (\%)=\frac{\frac{{D}_{IS}}{{S}_{r}}}{N}$	This study

Note: Equations in Table 1 may not be the exact original equations in the corresponding literature

*D* refers to the coverage (also called depth of coverage)

*R*_*i*_ refers to the aligned length of a read (sequence)* i* assigned to the reference gene/genome

*L* refers to lengths of reference genes/genomes

*m* refers to the mass (ng) of double-strand DNA, represented by sequenced reads in metagenome (DNA single-strands)

*An* refers to the count of adenine (A) in a metagenome

*Tn* refers to the count of thymine (T) in a metagenome

*Cn* refers to the count of cytosine (C) in a metagenome

*Gn* refers to the count of guanine (G) in a metagenome

*Sr* refers to the sequencing rate of DNA

*M* refers to the extracted DNA mass (ng)

*C* refers to the measured DNA concentration (ng/μL)

*V* refers to the volume of DNA elution (μL)

*SF* refers to the scaling factor

*N* refers to the number of IS added to samples, such as cell number of cellular IS [1, 21] or copies of DNA-IS [31], which has been normalized against the starting mass or volume of a sample

${D}_{IS}$ refers to the coverage of marker gene carried by cellular IS [1, 6, 21] or the number of reads assigned to DNA-IS [31]

*X* refers to the microbial load of a sample (also known as cell counts in unit mass or volume)

*A* refers to the relative abundance (%) of IS in amplicon sequencing dataset

*Q* refers to the total 16S rRNA gene copies measured by qPCR

*η* refers to DNA extraction efficiency. It is calculated as the ratio between the copies of IS in the extracted DNA, measured using qPCR (*N*_*D*_), and the copies of DNA-IS added to the sample (*N*), as shown in Eq. 7. Alternatively, *η* can also be calculated using the coverage of IS in the extracted DNA (i.e., the coverage of IS (*D*_*IS*_) normalized against the sequencing rate (*S*_*r*_)), divided by the number of IS added to the sample (*N*) in Eq. 8

#### 2.4 Summary and outlook

Each microbial load estimation method has specific application scenarios, and the method selection should depend on the physiochemical characteristics of the samples and the inherent requirements of the methods. For example, FCM may be preferred for well-dispersed water samples, whereas qPCR and dPCR could be more suitable for samples requiring high sensitivity and specificity, such as those from clinical settings. Integrating multiple techniques to enhance microbiome quantification is necessary, such as combining FISH and FCM could achieve the quantification and sorting of multiple microorganisms by targeting specific signals. Furthermore, the development of absolute quantification in a rapid and high-throughput manner will significantly benefit the decision-making process, such as in the timely assessment of microbial pollutants in environmental samples to safeguard public health.

### 3. IS-based absolute quantification

Environmental samples often contain highly aggregated microbial states with microbial cells clustering in flocs rather than being evenly distributed and complicated environmental matrices with interfering substances, which together diminish the feasibility of current methods for absolute quantification of microbial populations in diverse environmental habitats. Given the extensive experience of using the ISs in analytical chemistry, incorporating ISs as references in environmental samples is advantageous. ISs undergo processes concomitantly with indigenous populations from sample pre-treatment to data generation [6, 21] and function as an “anchor” to convert the relative abundance in sequencing datasets to absolute abundance. The principles underpinning the use of ISs for calibration to achieve AQ encompass (i) the capacity of the IS to capture variations introduced by different steps in the experimental workflow, i.e., sample pre-treatment, DNA extraction, library construction, sequencing [21, 45]; (ii) the assumption that throughout the workflow, the ISs behaves analogously to the native cells and/or DNA molecules in the sample (e.g., GC content, length, and primer binding affinity) [24, 42, 97]; and (iii) the ability to unequivocally identify the DNA-based-IS bioinformatically [1, 6], or to ensure that the ISs is exogenous from the studied biological samples [31, 98].

#### 3.1 Equations used in IS-based AQ

An IS can serve as an “anchor” for calculating the scaling factor (*SF*), which is the ratio between the number of ISs spiked in the sample per unit volume/mass and the number of ISs covered in the sequencing dataset, for example, marker-gene-based *SF*s (Eq. 4) [1, 21] (Table 1). Similarly, other parameters have been proposed, such as the size factor [98] and the IS normalization factor [99] for the downstream calibration. These factors are both sample- and IS-specific, facilitating the quantification of absolute abundance for a particular taxon and gene. The underlying assumption for using marker-gene-based *SF* is that one cellular IS cell carries one copy of the fluorescent marker gene [1, 21]. However, it has been noted that, under nutritious conditions, the ratio might surpass 1:1 due to the active and cell constant division [6]. As a result, the *SF* could be refined by introducing the adjustment factor, which is the ratio of between the coverage of marker gene and the coverage cellular IS genome for bacteria under exponential growth, and calculated the adjusted *SF* [6]. An alternative calculation is about to determine microbial load (*X*) by leveraging the relative abundance of the ISs in amplicon datasets (Eq. 5) [31]. If the DNA extraction efficiency (*ƞ*) can be ascertained, the absolute abundance of a specific lineage can be calculated via Eq. 6 (Table 1).

#### 3.2 IS selection and IS-based AQ applications

ISs can be classified into two categories: DNA-based-ISs, which are specific DNA molecules, and cellular ISs (cell-based-ISs), which are whole cells. DNA-based-IS encompasses genomic DNA (gDNA) from populations that are exogenous to the indigenous microbiome, and synthetic DNA (sDNA) sequences. For instance, Smet et al. (2016) pioneered the DNA-based-IS AQ method by investigating the feasibility of adopting gDNA from *Aliivibrio fischeri* (DSM 507, with a GC content of 38%, commonly associated with marine environments) and *Thermus thermophilus* (DSM 46338, with a GC content of 69%, typically found in geothermal environments) as DNA-based-ISs to study soil microbiomes [25]. To quantify microbial abundance in 16S and 18S rRNA gene copies per milliliters of seawater, the gDNA of *T. thermophilus* (representing the 16S rRNA gene IS) and *Schizosaccharomyces pombe* (representing the 18S rRNA gene IS) were spiked into cells on the filter membrane collected from marine water, followed by amplicon sequencing after the DNA extraction [100]. Given the influence of GC content on quantitative accuracy, Venkataraman et al. (2018) incorporated gDNA from two bacterial species, i.e., *Aliivibrio fischeri* and *Rhodopseudomonas palustris* with GC contents of 38% and 65% respectively, into fecal samples [101]. In addition, gDNA-based-ISs from a marine bacterium, *Marinobacter hydrocarbonoclasticus* (ATCC 700491, Gram negative, GC content of 57%), were introduced into the extracted DNA of dairy samples, followed by metagenomic sequencing, enabling the quantification of the absolute copy number of thousands of genes concurrently [99].

Besides, sDNA-based-ISs, which usually have two parts, i.e., conserved regions for universal primer binding and artificial regions for differentiating ISs from native sequences of indigenous populations [24, 96, 102, 103], have been utilized in a few environmental microbiome studies to compute the absolute abundance of amplicons. Taking into account common primer binding sites from genes used for the identification of prokaryotes (16S), eukaryotes (18S), and fungi (ITS), and sequence lengths, sDNA-based-ISs were directly added to soil samples, followed by DNA extraction, and amplification using universal PCR primers to calculate the absolute abundance of amplicon families (i.e., 16S, 18S, and ITS) [24]. Five sDNA-based-ISs were generated by referring to the most abundant bacterial and fungal populations in the studied environment and added in gradient concentrations to solid-state fermentation samples for liquor production to quantify absolute abundance of microbiota [103]. Maintaining the conserved regions of selected natural 16S rRNA genes from five microorganisms, Tourlousse et al. (2017) replaced the variable regions with artificial sequences to generate twelve sDNA-based-ISs and added them to calculate the abundance of microbes in sludge and soil samples [102]. The same strategy was adopted to design a single sDNA-based-IS with modification of a 45-bp region of a 733-bp segment from an *Escherichia coli* strain 16S rRNA gene, which was used to retrieve the concentrations of the 16S rRNA gene per gram of fecal sample [96]. Nine sDNA-based-ISs at different concentrations were mixed with a DNA pool of DNA extracted from the soil samples to quantify the absolute abundance of soil microbiome [104]. As an alternative, sDNA-based-ISs can be designed by referring to other feature sequences of whole genomes [105]. For instance, microbial genomes were retrieved from RefSeq, which represent features such as taxa, genome size, GC content, rRNA operon count, and isolated environments; subsequently, subsequences for each genome and inverted subsequences were selected, but the original genome features were retained to generate 86 sDNA-based-ISs with varying lengths (1–10 kb) and GC contents for absolute quantitative microbial profiling using metagenomes [106]. In these studies, in silico analyses were conducted to ensure that sDNA-based-IS sequences satisfy target the GC content ranges, lack homopolymers, have no repeats of > 16 bp and no self-complementary regions of > 10 bp [102, 103], and confirm the uniqueness of artificial sequences after conducting BLAST searches against NCBI databases [102, 103, 106].

However, DNA-based-IS cannot correct the cell lysis efficiency in DNA extraction; thus, the cellular IS is suggested. The rationale of selecting a cellular IS is based on the assumption that the extraction efficiencies and sequencing biases of endogenous microbes are the same as those of the selected cellular ISs. Additionally, the cellular ISs should either be absent from the studied environment or can be clearly distinguishable from endogenous microorganisms. Stämmler et al. (2016) spearheaded the adoption of three exogeneous bacterial strains (i.e., *Salinibacter ruber* (Gram negative), *Rhizobium radiobacter* (Gram negative), and *Alicyclobacillus acidiphilus* (Gram positive)) as cellular ISs to quantify the bacterial absolute abundance of gut specimens [98]. The usage of an environmental bacterium, *Sporosarcina pasteurii* (Gram positive), as a cellular IS together with replicate sampling to achieve absolute quantification of microbial populations in soil and stool samples to evaluate the reproducibility revealed that more than 50% of species changes is due to technical variations, such as in the sampling step [31].

Besides, engineered microbes with marker gene labels can be used as cellular ISs when the marker gene is absent from the studied environment and is distinguishable from genes of indigenous populations. Using marker-gene labeled cellular ISs has two additional assumptions: (i) the genome coverage of the cellular ISs is the same as the coverage of the single-copy marker gene or at a fixed ratio; and (ii) the mapping recovery rates of different genes/populations are the same as those of the marker gene. The cellular IS-based method was developed using *mClover*-labeled *E. coli* with nanopore sequencing to achieve rapid identification and quantification of pathogens and ARGs in influent and effluent samples from wastewater treatment plants (WWTPs) [1]. Considering the inconsistent DNA extraction efficiencies between Gram positive and negative microbes, *mCherry*-labeled *Bacillus amyloliquefaciens* (Gram positive) and *gfp*-labeled *Pseudomonas putida* (Gram negative) were applied as cellular ISs with Illumina sequencing to profile the population dynamics under various anaerobic digestion operating conditions [21]. The cellular IS-based AQ application was broadened from documenting the absolute abundance of microbial populations to quantitative microbial risk assessment (QMRA) for beach water using *egfp*-labeled *Pseudomonas_E hunanensis* via nanopore sequencing [6].

The accurate identification and quantification of ISs have been demonstrated via mock communities which have known amounts, as their quantitative results closely align with pre-designated values [102, 106], which validates the feasibility of adopting ISs in diverse environmental samples. For example, when sDNA-based-ISs were added to a plasmid-based DNA mock community of 15 bacterial strains at known concentrations, the resulting proportion was 35.8 ± 4.2% (range 31.6–41.0%), which is comparable to the expected value of 44.4% [102]. A staggered mixture of sDNA-based-ISs was incorporated into gDNA from a mock community at an abundance of 1%, and a value of 1.5% was observed [106].

### 4. Addressing uncertainties of IS-based AQ with a technical checklist

**Fig. 3:**
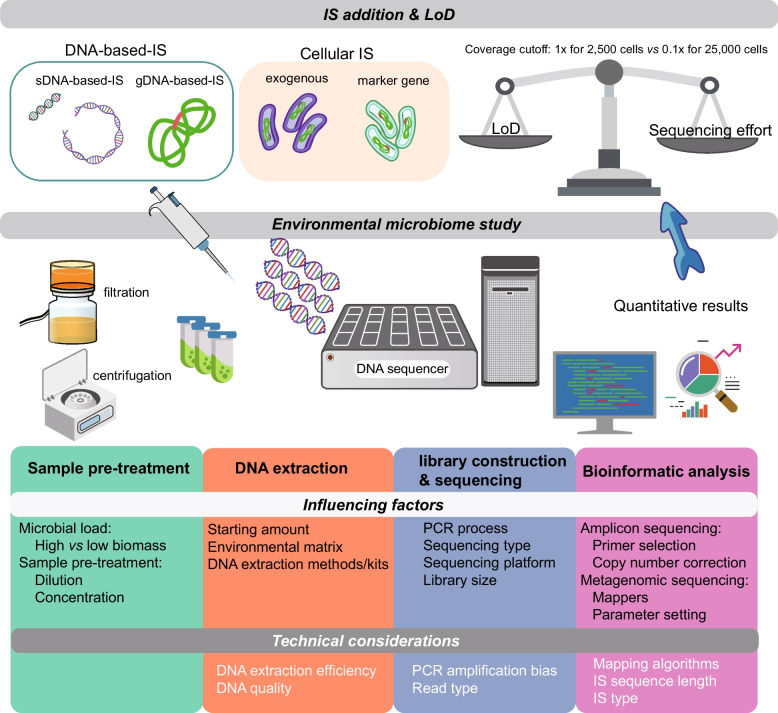
Influencing factors and technical considerations for a reporting checklist in IS-based AQ methods

It is crucial to pose critical questions that can guide the selection of the most suitable AQ method for an environmental microbiome study. One should assess whether the cells within samples are well suspended or in a heterogeneous state. If cells are well suspended, direct counting could be used together with ISs for a double check, while, in principle, the IS already can capture technical variations in multiple-step analyses [45]. A systematic approach involving a checklist and technical suggestions can aid in identifying the optimal strategy for using an IS-based AQ (Figure S1 and SI 1). The key factors to consider in the experimental design include determining the most suitable IS type (e.g., DNA-based-IS or cellular IS), assessing the optimal quantity of ISs to be incorporated into the samples, and evaluating the lowest concentrations that can be reliably discerned. Additionally, it is also important to ascertain at which processing step the IS should be spiked, select the sequencing approach (i.e., metagenomic or amplicon), and validate the accuracy and reliability of the chosen method (Fig. 3 and Table S2).

#### 4.1 Optimizing IS addition for accurate quantification

A positive correlation has been observed between the IS input and the total number of read counts assigned to the IS in both amplicon and metagenomic sequencing datasets [98, 99, 102, 103]. This relationship has been substantiated in multiple studies, including those utilizing staggered IS mixtures (of 12 sDNA-based-ISs) with a dynamic range of approximately 2^10^ [102], inputting IS concentrations varying across three orders of magnitude, i.e., spike-in mass percentage (ratio of mass between spiked-in DNA/total DNA) of 0.1%, 1%, and 10% [99], and employing sDNA-based-ISs at five distinct concentrations, ranging from 10^4^ to 10^8^ copies/g solid fermentation samples [103].

However, using low IS amounts may lead to biased quantitative results [24], whereas using high IS amounts will scarify the effective sequencing dataset size. As benchmarked by a *Rhizobium* pure culture, accurate estimates of *Rhizobium* abundance were achieved with high levels of IS addition, whereas low levels led to substantial underestimation [24]. Moreover, a dilution series experiment revealed a more robust linear relationship between soil weight and total 16S rRNA gene copies quantified using gDNA-based-IS AQ when gDNA-based-ISs were added at 1% rather than at 0.1% of total DNA [25]. A study using three gDNA-based-IS addition percentages (0.1%, 1%, and 10%, IS DNA/total DNA mass) suggested that IS addition should exceed 0.1% to ensure the detection of ISs in metagenomic sequencing [99]. These findings underscore the importance of optimizing IS addition to environmental samples, as it is crucial for ensuring accurate detectability [45, 99] while efficiently allocating sequencing resources to microbes in samples [24, 25, 45, 106, 107].

#### 4.2 Correcting variability in DNA extraction

The variability in DNA extraction across samples can be attributed to differences in environmental matrices, microbial populations, extraction methods/kits, etc. Considering the high diversity and heterogeneity of microbial populations in environmental samples, it is suggested to use a larger starting amount of sample within the recommended mass or volume range specified in the protocol, to achieve an increased DNA amount for better presentiveness, on top of replicate samples and homogenization pre-treatment.

The DNA extraction process typically comprises three main steps: cell lysis, DNA isolation from cell lysates, and DNA purification. Sometimes, concentration is required for low-biomass samples. Generally, DNA extraction efficiency (*η*) is calculated by using spiked cellular ISs or DNA-based-ISs (not simulating cell lysis), and by comparing the detected values and the known spiking amount of ISs [96, 103]. When sDNA-based-IS was spiked into soil samples, the *η* were 40 ~ 84% of soil samples using Quick-DNA™ Fecal or Soil Microbe Miniprep Kit™ [96], and 72 ~ 87% of the bacteria in a community using E.Z.N.A. soil DNA kit [103].

To include the cell lysis efficiency in calculating DNA extraction efficiency, cellular ISs are more acceptable under the assumption of the consistency of extraction efficiency between cellular IS cells and indigenous cells of environmental samples. The *η* were 75 ~ 110% using QIAamp PowerFecal kit for manure slurry and manure stockpile samples [99]. There are two ways to calculate the *η*. One method is to take the ratio between the copies of the IS in the extracted DNA, measured using qPCR, and the copies of the ISs added to the sample, as shown in Eq. 7. Alternatively, *η* can also be calculated using the coverage of the ISs in the extracted DNA (i.e., the coverage of the ISs normalized against the sequencing rate), divided by the number of the ISs added to a sample (Eq. 8).

#### 4.3 LoD in absolute quantification using metagenomic sequencing

The LoD serves as a crucial indicator of AQ methods, with a lower LoD indicating increased sensitivity in AQ methods. However, the LoD of AQ methods based on metagenomic sequencing is subject to the influence of various factors such as sequencing depth [1, 99] and microbial loads [106]. An increase in the LoD, i.e., lower sensitivity, was observed when the sequencing depth decreased or the microbial load of the sample increased [1, 21]. It is essential to recognize that LoD is also species-specific due to different genome sizes [21].

There are inconsistent definitions of LoD across different studies. One study added gDNA-based-ISs into the extracted DNA of samples at three gradient percentages (i.e., 0.1%, 1%, and 10%) and sequenced at a depth of 50 million 150 bp paired-end reads of each metagenome [99]. LoD was ascertained as 3.2 × 10^7^ gene copies/g (dry weight) sample of dairy waste, since 95 out of 4272 genes of the ISs cannot be detected at 0.1% IS addition percentage [99]. Indeed, higher coverage of genes/genomes in AQ methods (Eq. 1) ensures higher reliability of absolute quantitative results [106]. However, setting the coverage cutoff too high may result in unnecessary conservative LoD, which will underestimate the sensitivity of the method. For example, the LoD defined via the criterion in the above study, which requires that the majority of genes on the genome of a species should have at least 1 × coverage [99], is very conservative one compared with another criterion, which only requires 1 × coverage of a region in any of the unique genes of microbial genomes [1, 21].

Our previous studies determined the LoD by explicitly specifying the minimum read length assigned to a particular taxon/gene [1, 6, 21]. With a minimum nanopore read mapping length of 1 kb as the cutoff of a prokaryotic taxon, the LoDs of *Klebsiella pneumoniae* were 19 cells/mL and 591 cells/mL at sequencing depths (sequencing data amounts) of 12.6 Gb and 0.57 Gb, respectively, in the influent sample (with a microbial load of 3.43 × 10^11^ cells/L) from sewage treatment works [1]. When 150 bp was used as the minimum Illumina read mapping cutoff, the developed AQ method reported LoDs of 131 ± 94 cells/mL and 265 ± 136 cells/mL for Gram-negative microbes and Gram-positive ones, respectively, in anaerobic digestion of sewage sludge at a sequencing depth of 10 Gb [21]. We suggest that the LoD (in the unit of cells or copies per unit volume or mass) should be calculated using the minimum alignment length of metagenomic reads assigned to specific taxon/genes, normalized genome size or gene length, and converted to absolute abundance using *SF*s, in metagenomic studies using IS-based AQ methods. The LoD using the same approach will be different for samples with various microbial loads. Interestingly, this LoD is independent of the amount of ISs addition. Microbial loads and genome size/gene length cannot be controlled, while the sequencing depth is another crucial factor affecting LoD, and striking a balance between sequencing effort and LoD constitutes another critical technical consideration, particularly in metagenomic sequencing.

#### 4.4 Enhancing AQ with multiple ISs and using ISs to calibrate variability

Multiple ISs can benefit the AQ in three aspects. First, compared with the use of a single IS, the adoption of multiple ISs can provide insights into variations inherent to sequences or cells that emulate a variety of taxa [102], different gram-stain phenotypes (such as cell wall structure) [6, 21], genomes of varied GC contents [102, 106], cell shapes, etc. Second, by benchmarking known ratios between multiple ISs, potential outlier ISs can be easily identified and omitted from the subsequent calibration [45, 108]. Third, the recovery rates of different ISs have been shown to vary largely due to their differential detection efficiencies. Multiple ISs can be used to “average-out” differential detection rates to normalize the technical noise [102] or sum the read counts of all ISs for the calibration to reduce errors [98]. Multiple ISs can be employed in two ways: (i) all ISs are mixed approximately equally [6, 21]; or (ii) they are staggered mixtures with a wide range of concentrations of ISs [102, 106].

Bias could be introduced in different steps of microbiome studies, from sample pre-treatment to DNA extraction, library construction, sequencing, and bioinformatic analyses (Fig. 3). Like cellular ISs, DNA-based-ISs can be spiked at different steps to benchmark variations across various technical stages. When added to soil samples [25, 96], to human fecal samples [101], to seawater samples [100], and liquor solid-fermentation samples [103] before DNA extraction, DNA extraction efficiencies can be calculated although DNA-based-ISs do not simulate cell lysis. Even just spiking DNA-based-ISs into DNA pools extracted from manure-related samples [99], soil [104], and saltmarsh samples [106] allows for benchmarking the technical variations caused by library construction and sequencing for better quantification. 

#### 4.5 Validation of IS-based AQ

The feasibility of most IS-based AQ methods is substantiated by employing mock communities or alternative independent enumeration techniques. Among the sixteen IS-based AQ investigations, seven utilized mock communities to corroborate the accuracy of the quantitative results generated by the suggested methodologies. These include the incorporation of commercialized ZymoBIOMICS™ microbial community standards [1, 6] and laboratory-made artificial communities, such as one consisting of 16 bacteria and fungi at equal cell concentration [101], gDNA from a well-defined MBARC-26 mock community [106], plasmids containing near-full-length 16S rRNA genes of 15 different bacteria [102], a microbial community consisting of five bacteria and five fungi [103], and a *Rhizobium leguminosarum* pure culture cell suspension [24]. In conjunction with mock communities, various enumeration methods have been employed to assess the feasibility of IS-based AQ methods, such as qPCR [24, 98, 99, 102, 103], direct counting using FCM [21, 100], and indirect estimation using the PLFA [25].

In addition to the validation of the accuracy of IS-based AQ methods, mock communities and ISs could also be used to identify uncertainties in bioinformatic analysis, especially when using metagenomic sequencing. Mapping the reference ISs for the quantification will be influenced by multiple factors, such as sequencing read types, mappers and parameter settings, reference gene length, reference gene GC contents, and nonspecific mapping [1, 99, 106]. With respect to the evaluation of mapping algorithms, Bowtie2 (paired mode) outperformed Kallisto when Illumina reads were used, as determined by the gene recovery of the spiked gDNA [99]. Consequently, an 80% identity threshold was employed using nanopore sequences with Minimap2 (map-ont) as the mapper [6], and the 99% identity threshold was deployed using Illumina reads with the mapper of Bowtie2 (paired mode) [21] for mapping the fluorescence marker gene *mCherry*. Additionally, reference IS recovery may be underestimated when the gene length is near or shorter than the library insert size, since read pairs are less likely to map accurately because a substantial portion of the read pair extends beyond the target gene reference sequence [99]. Furthermore, DNA-based-IS recovery decreased with increasing GC content and then gradually increased using HiSeq4000 platform [99]. The mismatch error rate of each sDNA-based-IS steadily increased with increasing GC contents, but limited GC bias was observed in the coverage of reference genes using PCR-free nanopore sequencing [106]. GC bias in DNA sequencing library preparation and sequencing platforms has notably been reported, especially from MiSeq, NextSe, followed by Nextera XT, PacBio, and HiSeq sequencing platforms [109]. When gDNA is used as ISs, nonspecific mapping can impact the mapping process and distort the quantification results [99]. In this situation, more mapping tools with algorithms developed under different assumptions should be tested, and stricter mapping parameter cutoffs, such as higher alignment identities and coverage thresholds, are recommended. Obviously, in such instances, sDNA-based-ISs can alleviate bias caused by nonspecific mapping [106].

#### 4.6 Metagenomics vs. amplicon sequencing

Both metagenomic and amplicon sequencing can be integrated with ISs to achieve AQ for microbial populations and/or elements in environmental samples. These two sequencing technologies possess distinct advantages and limitations and offer specialized insights into microbial communities.

Compared with metagenomic sequencing, inherent PCR amplification biases in amplicon sequencing can distort quantitative results. First, biases attributed to varying templates of different GC contents have been observed [100], as templates with higher GC contents have higher melting temperatures and are less efficiently amplified [92, 94]. Second, amplification bias is introduced by primer sequence selection [91, 93] and the number of PCR cycles [110], as DNA sequences with G/C at the degenerate position can be overamplified compared with sequences with A/T [100]. Third, the PCR process is influenced by sample characteristics, such as low biomass samples are prone to bias caused by overamplification, and as the number of PCR cycle increases, contaminating microorganisms are cumulatively over-represented [19, 41]. If amplicon sequencing is adopted, ISs should be designed according to the primers, GC contents, and sequencing length corresponding to the amplification regions [24, 96, 102, 103] and tested with different primer sets [102]. The necessity of correcting the 16S rRNA gene copy number into cell number is a matter of debate. Caution should be exercised in rRNA gene copy number correction, as a significant portion of operational taxonomic units (OTUs) or amplicon sequence variants (ASVs) remains unclassified, and the limited number of known 16S rRNA gene copy numbers from sequenced genomes likely does not reflect the natural variability in 16S rRNA gene copy numbers [100]. Additionally, existing tools perform poorly for the majority of tested genomes and OTUs [111].

Metagenomic sequencing has been reported to be less susceptible to PCR bias [42, 99, 106] and allows for the profiling of the full-spectrum of genes in an entire community [112], transcending the limits of phylogenetic marker gene analysis [41]. Additionally, owing to the ongoing improvement in chemical and sequencing technologies, significant progress has been made in long-read nanopore and PacBio sequencing. However, while there are inherent problems with the sensitivity and specificity of mapping algorithms, challenges also arise from the relatively high error rate in nanopore long reads during quantification. Additionally, the quality of DNA is crucial when nanopore sequencing is employed. For instance, it has been suggested that the DNA purity should meet the requirements of an A260/A230 ratio > 2 and an A260/A280 ratio > 1.8 for human gut microbiome studies [113]. Researchers should carefully review and modify existing DNA extraction and purification protocols prior to nanopore sequencing to ensure optimal sequencing results.

#### 4.7 Summary and outlook for IS-based AQ methods

Current practices advocated for standardizing microbiome studies include adopting consistent storage approaches and pre-treatment methods, following identical experimental protocols, and employing the same analytical tools. Quantifying microbial populations in absolute abundance has biological meaning and provides more comparable, reliable, and reproducible datasets. Arguments about the poor representativeness of cellular ISs for indigenous microbial populations highlight the need to develop more engineered cellular ISs. By leveraging our knowledge of the phylogeny of microbes in the studied environment, we can genetically modify these species by adding marker genes to their genomes and use marker gene labeled engineered cells as cellular ISs, thereby simulating indigenous microbes. This approach shares the same experimental design for quantitative stable isotope probing, which is crucial for the identification and quantification of active microbial populations under given conditions [114, 115]. Moreover, marker-gene-labeled engineered archaeal populations are essential, as they play critical roles in diverse environments and biochemical cycles. To increase the reliability and applicability of IS-based AQ methods, incorporating a checklist of technical factors in the method’s development and application processes is of highly importance (SI 1 and Figure S1). Before IS application, ISs with gradients should be sequenced together with real samples to establish a relationship between spiked amounts and quantified values. Important actions include spiking cellular ISs during sample collection or pre-treatment to capture variations inherent in the entire methodological workflow, employing multiple ISs, optimizing IS addition amounts, and validating the method with microbial mock communities or independent enumeration approaches. Furthermore, metagenomics offers a broader scope of information than amplicon sequencing does, especially as the ongoing development of long-read sequencing technologies overcomes the limitation of short reads. However, meticulously addressing biases stemming from bioinformatics analyses, which could be evaluated and partially resolved using simulated datasets, is crucial.

### 5. Application opportunities of EAM

**Fig. 4:**
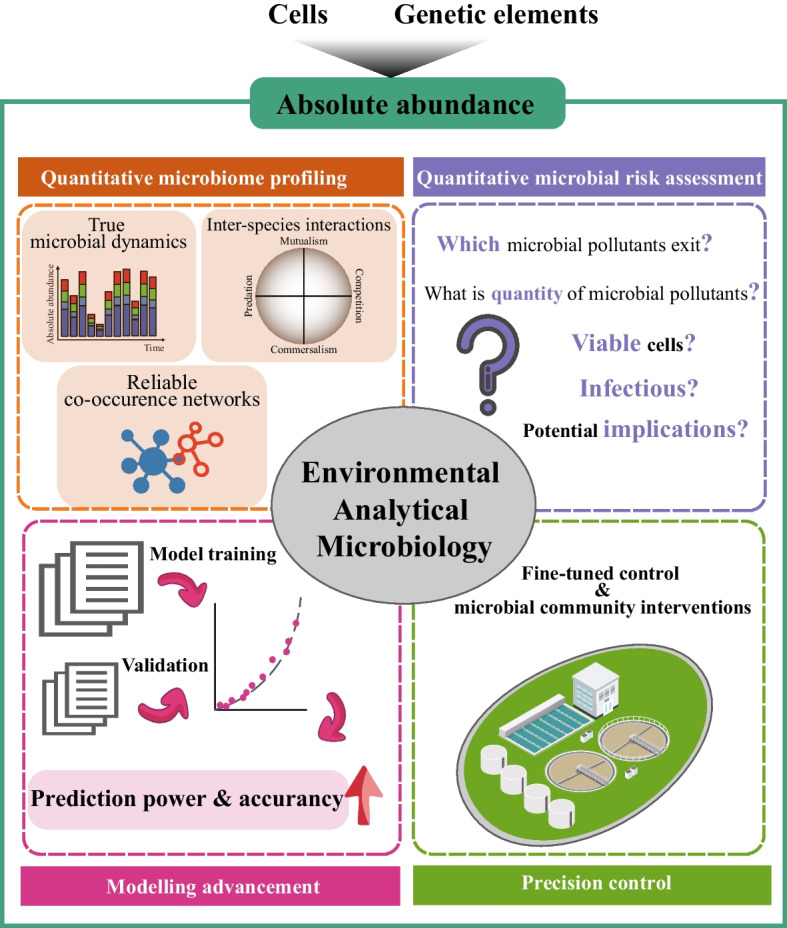
Applications using absolute quantitative microbiome in environmental analytical microbiology

The field of EAM has advanced to enable the detection of even trace amounts of microbial pollutants, such as pathogens and ARGs. By utilizing absolute quantitative metagenomics, this scientific discipline allows for the identification of microbial pollutants across diverse systems and establishes links between targeted ecological or engineered systems and key microbial populations or pollutants. As a result, such knowledge lays the foundation for the development of precise control strategies in various systems through the deliberate manipulation of microorganisms (Fig. 4).

#### 5.1 Quantitative microbiome profiling

Quantitative microbiome profiling circumvents compositionality effects by documenting microbial dynamics in absolute values [18]. This approach enables the reconstruction of reliable co-occurrence networks of microorganisms, the identification of genuine inter-species interactions, and the provision of insights into underlying ecological laws in response to varying disturbance variables. For example, the absolute quantitative metagenomics allows the calculation of the growth and decay rates of microbes under different living conditions [21, 26].

#### 5.2 Quantitative microbial risk assessment

Key questions that must be addressed by QMRA include the following: (i) Which microbial pollutants exist, pathogens and/or ARGs? (2) What is their quantity? (3) Are pathogens viable? (4) Do ARGs have high dissemination potential [116]? The insights gained from QMRA are crucial for setting standards of microbial regulatory parameters and legislative frameworks.

IS-based methods in analytical chemistry are used to develop standard methods, such as the U.S. EPA Method 200.8 [117], which is employed for regulatory monitoring of trace elements in drinking water and wastewater in compliance monitoring programs, such as the Clean Water Act and the Safe Drinking Water Act. IS-based analytical methods, such as the U.S. EPA Method 1694 [118], which is a standard method developed to screen samples from various sources to profile the occurrence and concentrations of pharmaceuticals and personal care products, are used to detect organic pollutants. However, standard methods used for microbial pollutants are primarily cultivation-based, such as methods for enumerating cells of fecal coliforms or *E. coli* [119]. The selection of *E.coli* as a regulatory parameter of public health significance hinges on the availability of a standard analytical method [119], underscoring the necessity for standardized methods for microbiome studies. The WHO has published an updated list of bacterial priority pathogens, including 24 bacteria across 15 families of antibiotic-resistant pathogens [120]. High-throughput output and wide-spectrum screening capabilities are additional needs of standard methods. As sequencing becomes prevalent in microbiome research and as genomic analysis matures into becoming more quantitative, IS-based absolute quantitative metagenomics enables the generation of reliable, comparable, systematic, and reproducible datasets. These datasets can be applied to establish regional and global baselines and to conduct QMRA. In turn, this process can facilitate the formulation of regulatory standards and legislative frameworks to safeguard the total environment.

#### 5.3 Improvement of mathematical modeling

Mathematical modeling facilitates the comprehension of complex ecological interactions, including how microbial populations respond to environmental changes, how inter-species interactions affect community dynamics, and how microbial metabolic traits contribute to biogeochemical cycles and system stability [1]. By incorporating the absolute abundance of individual taxa to train models, the accuracy and predictive power of models can be greatly enhanced. Furthermore, the integration of multi-omics data in absolute abundance values, such as metatranscriptomics and metabolomics, offers a multi-layer perspective on microbe-mediated systems by capturing the interplay between community composition (community structure), gene expression, and metabolic activities in response to different environmental variables. This multidimensional approach promotes the development of more advanced mathematical models.

#### 5.4 Precision control in engineered systems and microbial community interventions

By adopting absolute abundance data, the removal efficiencies of microbial pollutants in sewage treatment work can be calculated [1], and the specific activity of functional microorganisms can be computed to evaluate the performance of engineered systems [21]. Documenting the dynamics of microbes serves as a crucial reference for fine-tuned manipulation of complex communities by simplifying or deconstructing them using a drop-out approach after key environmental variables that promote the enrichment of functional microbes are identified [121–123]. Moreover, employing absolute abundance information in the design process of engineered systems enables a better representation of desired microbial populations, ultimately leading to improved system performance and a more tailored approach to address specific environmental and biological challenges.

## 6. Conclusion

The rapid increase in studies on the environmental microbiome using high-throughput sequencing has facilitated the development of EAM. Nonetheless, bias in different steps of sequencing-based EAM and compositional data can compromise the reliability of quantitative results and impede effective comparisons. To address these challenges, quantifying microbial elements in absolute abundance using ISs is crucial for standardizing microbiome analyses. The estimation of total microbial load and DNA mass-based metagenomic absolute quantification approaches can be utilized for AQ, although inherent limitations of quantification methods restrict their application. To capture technical variability of multiple-step microbial analysis, IS-based AQ methods are recommended as they provide both quantitative and qualitative data, while also serving as quality control. However, uncertainties in IS-based AQ methods should be properly resolved to achieve reliable comparisons across samples and studies. In principle, EAM using an absolute quantitative microbiome enables applications such as microbial co-occurrence network reconstruction and risk assessment, contributing to a deeper understanding of microbial interaction and effective management strategies. Integrating multi-omics data and tracking microbial taxa dynamics also facilitates advanced model development and improved performance of engineered systems.

## Supplementary Information

Supplementary Material 1. 

## Data Availability

No datasets were generated or analysed during the current study.
