# Supplementary material for: The quest for environmental analytical microbiology: absolute quantitative microbiome using cellular internal standards
Source: Microbiome. 2025 Jan 27;13:26. doi: 10.1186/s40168-024-02009-2 (PMC11773863; doi:10.1186/s40168-024-02009-2)
Supplement: Supplementary file 2 — Supplementary Material 1. [file 40168_2024_2009_MOESM1_ESM.docx]

**Supporting Information**

**The Quest for Environmental Analytical Microbiology: A****bsolute Quantitative Microbiome using Cellular Internal Standards**

Chunxiao Wang ^a^, Yu Yang ^a^, Xiaoqing Xu ^a^, Dou Wang ^a^, Xianghui Shi ^a^, Lei Liu^a^, Yu Deng ^a,e^, Liguan Li ^a,f^, Tong Zhang ^a,b.c,d^ *

Affiliations

^a^ Environmental Microbiome Engineering and Biotechnology Laboratory, Center for Environmental Engineering Research, Department of Civil Engineering, The University of Hong Kong, Pok Fu Lam, Hong Kong, China

^b^ School of Public Health, The University of Hong Kong, Hong Kong, China

^c^ Macau Institute for Applied Research in Medicine and Health, Macau University of Science and Technology, Macau SAR, China

^d^ State Key Laboratory of Marine Pollution, City University of Hong Kong, Hong Kong SAR, China

^e^ Division of Applied Oral Sciences & Community Dental Care, Faculty of Dentistry, The University of Hong Kong, Hong Kong, China

^f^ Department of Science and Environmental Studies, The Education University of Hong Kong, 10 Lo Ping Road, Tai Po, New Territories, Hong Kong, China

*Corresponding author.

Email: [zhangt@hku.hk](mailto:zhangt@hku.hk)

Phone: +852-28578551. Fax: +852-25595337.

**SI 1 The technical checklist and recommendations in the IS-based AQ study**

The IS selection and addition amount are primarily determined by three factors: 1) research objectives, 2) sample characteristics and 3) microbial load (Figure S1). Using multiple ISs is recommended, as they can capture more technical variations than a single IS can capture. When the research objective is to quantify the absolute abundance of microbial populations and/or cellular genetic elements, cellular ISs are preferable over DNA-based-ISs. Conversely, DNA-based-ISs are suitable for direct addition to environmental samples if the research focuses on quantifying extracellular DNA. When exogenous microbes are chosen as cellular ISs or using their genomic DNA (gDNA) is used as gDNA-based-ISs, it is essential to pre-screen the signals of ISs from the studied environmental microbiome to ensure that there is no inherent noise would distort subsequent quantitative results. If engineered microbes with marker genes are selected as cellular ISs, the quantitative results for phylogenies closely related to these cellular ISs should be interpreted with caution owing to potential biases introduced by bioinformatic analyses.

The amount of IS added is determined by the total microbial load, with a recommended range of 1%-5% of total microbial load. This range balances the limit of detection (LoD) and the sequencing effort. Adding cellular ISs at the beginning of a microbiome study, such as at sample pre-treatment, is recommended, as it allows for the correction of biases generated from multiple-step microbial analyses. Using cellular ISs to mitigate the influence of sample pre-treatment on quantitative results is particularly useful, as pre-treatments are commonly needed. High-biomass samples may require dilution, and low-biomass samples may need the volume reduction.

The inconsistency of DNA extraction efficiencies across diverse samples hinders inter-sample comparison. Therefore, we suggest adding cellular ISs prior to DNA extraction. Evaluating DNA extraction performance across various protocols and kits, with a focus on DNA quantity and purity, is vital. Selecting the best performed extraction methods/kits and modifying extraction procedures are essential for yielding high-quality DNA, this is crucial for the success of library construction and sequencing, particularly when Nanopore sequencing is used.

Amplification bias is a major concern in amplicon sequencing, hence, the use of multiple ISs covering a wide range of GC contents is suggested. The LoD for IS-based AQ method using amplicon sequencing can be defined as at least one quality-controlled reads assigned to a specific taxon. Another key factor to consider is the variation in gene copy number across different microbial taxa, and there is ongoing debate about the necessity of gene copy number correction. For metagenomic studies, the technical evaluation should be conducted at the bioinformatic analysis step, especially when dealing with different read types, such as long reads and short reads. Different mappers and their respective parameters should be evaluated, which can be benchmarked with simulated datasets. Notably, we suggest the use of a mock community comprising known cell count populations to validate the developed IS-based AQ method before its application.

Table S1 Overview of methods used for estimation of total microbial load in environmental microbiome.

| **Group** | **Methods** → **Target** | **Advantages** | **Shortcomings** | **Influencing factors** | **Ref.** |
| --- | --- | --- | --- | --- | --- |
| Direct counting methods | Heterotrophic plate count and multiple-tube fermentation technique  → Cultivable microbe | - Well-established protocol. | - Time-consuming; - Labor-intense; - The need of sample dilution or concentration; - Underestimation of total microbial loads, due to cells are unculturable, viable not non-culturable, or dead. | - Culture conditions, e.g., culture media, temperature, etc.; - Cell activity and varied growth rates; - Cell distribution status. | [1-5] |
|  | Epifluorescence microscopy and Hemocytometer  → Total cells | - Well-established protocol. | - Labor-intense; - The need of sample dilution or concentration; - Sensitive to operator’s skills; - Sensitive to cell distribution status. | - Cell distribution status; - Operational skills, e.g., even cell distribution during the filtration, the selection of representative areas using microscope, etc. | [6-9] |
|  | Flow cytometer  → Total cells | - Highly informative nature; - Rapid and automatic processing; - High accuracy and reproducibility; - Cost-effectiveness. | - No universal FCM setting and analytical protocol; - Sensitive to cell distribution status; - Sample pre-treatment is required, especially for complex samples. | - Operational skills and operator’s experience; - Cell distribution status. | [10-15] |
| Indirect microbial biomass estimation | UV-vis spectrophotometric optical density (OD)  → Total cells | - Rapid and reliable for pure culture. | - The need of a standard curve relating actual cell counts and OD values. |  | [16, 17] |
|  | Volatile suspended solids  → Biomass | - Well-established protocol. | - Imprecise due to the presence of organic particles in VSS. | - The presence of organic particles. | [18-20] |
|  | Total DNA amount  → Biomass | - Well-established protocol. | - Limited references; - Imprecise due to the presence of DNA originating from non-bacterial (e.g., fungal, viral, protozoan) sources. | - Various genome size of diverse microbes; - DNA extraction efficiency. | [21] |
|  | Microbial biomass carbon and nitrogen (MBC & (MBN); phospholipid fatty acid (PLFA); Adenosine triphosphate (ATP) → microbial indicator |  | - Limited references; - Need calibrations between cell numbers and values of MBC, MBN, PLFA, and ATP values. | - Matrix effect. | [22-28] |
| Molecular methods | qPCR; dPCR → amplicons | - High sensitivity and specificity; - High-throughput; - Large detection ranges; - Well-established protocol (qPCR); - Cost-effectiveness (qPCR); - Rapid turnaround time; - No need of a standard curve (dPCR); - Tolerance to sample matrices (dPCR). | - Bias due to varied DNA extraction efficiencies; - Contamination issue, especially for low microbial load samples; - The need of pre-evaluation on designed primers/primer-probe sets/qPCR assay; - Amplification bias; - The need of a standard curve (qPCR); - Sensitive to matrix effect of samples (qPCR). | - DNA extraction efficiency; - Matrix effect; - Operational skills. | [29-41] |

Table S2 Technical details of IS-based AQ studies.

| **IS type** | **IS feature** | **No.**  **of IS** | **Sample type** | **Validation strategy**  **(Mock community**  **& Other method)** | **IS spiking step** | **Ref.** |
| --- | --- | --- | --- | --- | --- | --- |
| Cellular IS | Exogenous | 3 | Feces | qPCR | Add to samples before DNA extraction | [42] |
| Cellular IS | Exogenous | 1 | Soil and feces | N.A. | Add to samples before DNA extraction | [43] |
| Cellular IS | Exogenous | 3 | Soil | N.A. | Add to samples before DNA extraction | [44] |
| Cellular IS | Maker gene | 1 | Influent and effluent samples from WWTPs | ZymoBIOMICS^TM^ microbial community standard II &  Selective HPC on *﻿Klebsiella* spp. | Add to samples before DNA extraction | [45] |
| Cellular IS | Maker genes | 2 | Anaerobic digested slurry | FCM | Add to samples before DNA extraction | [46] |
| Cellular IS | Maker gene | 2 | Beach water | ZymoBIOMICS^TM^ Gut Mock Community &  Selective HPC on *﻿E. coli* and *Vibrio* spp. | Add to samples before DNA extraction | [47] |
| gDNA-based-IS | Exogenous | 1 | Soil | PLFA/SIR | Add to samples before DNA extraction | [48] |
| gDNA-based-IS | Exogenous | 2 | Feces | Mock community of 16 microbes at equal cell number | Add to samples before DNA extraction | [49] |
| gDNA-based-IS | Exogenous | 2 | Sea water | FCM | Add to samples before DNA extraction | [50] |
| gDNA-based-IS | Exogenous | 1 | Dairy manure slurry | ZymoBIOMICS Spike-in Control I ^a^ & qPCR | Add to extracted DNA | [51] |
| sDNA-based-IS | Synthetic | 86 | Saltmarsh soil | MBARC-26 genomes (even mixture and staggered mixture) | Add to extracted DNA | [52] |
| sDNA-based-IS | Synthetic | 12 | Soil and sludge | Mock community & qPCR | Add at DNA step after cell lysis | [53] |
| sDNA-based-IS | Synthetic | 3 | Soil | *Rhizobium leguminosarum* cells & qPCR | Add to samples before and after DNA extraction | [54] |
| sDNA-based-IS | Synthetic | 1 | Feces | qPCR | Add to samples before DNA extraction | [55] |
| sDNA-based-IS | Synthetic | 5 | Solid-state fermentation samples | Mock community & qPCR and Microscopy | Add to samples before DNA extraction | [56] |
| sDNA-based-IS | Synthetic | 9 | Soil | N.A. | Add to extracted DNA | [57] |

Note: ^a^ the mock community is used to estimate the DNA extraction efficiency between the spiked Gram-positive and Gram-negative microorganisms.

**
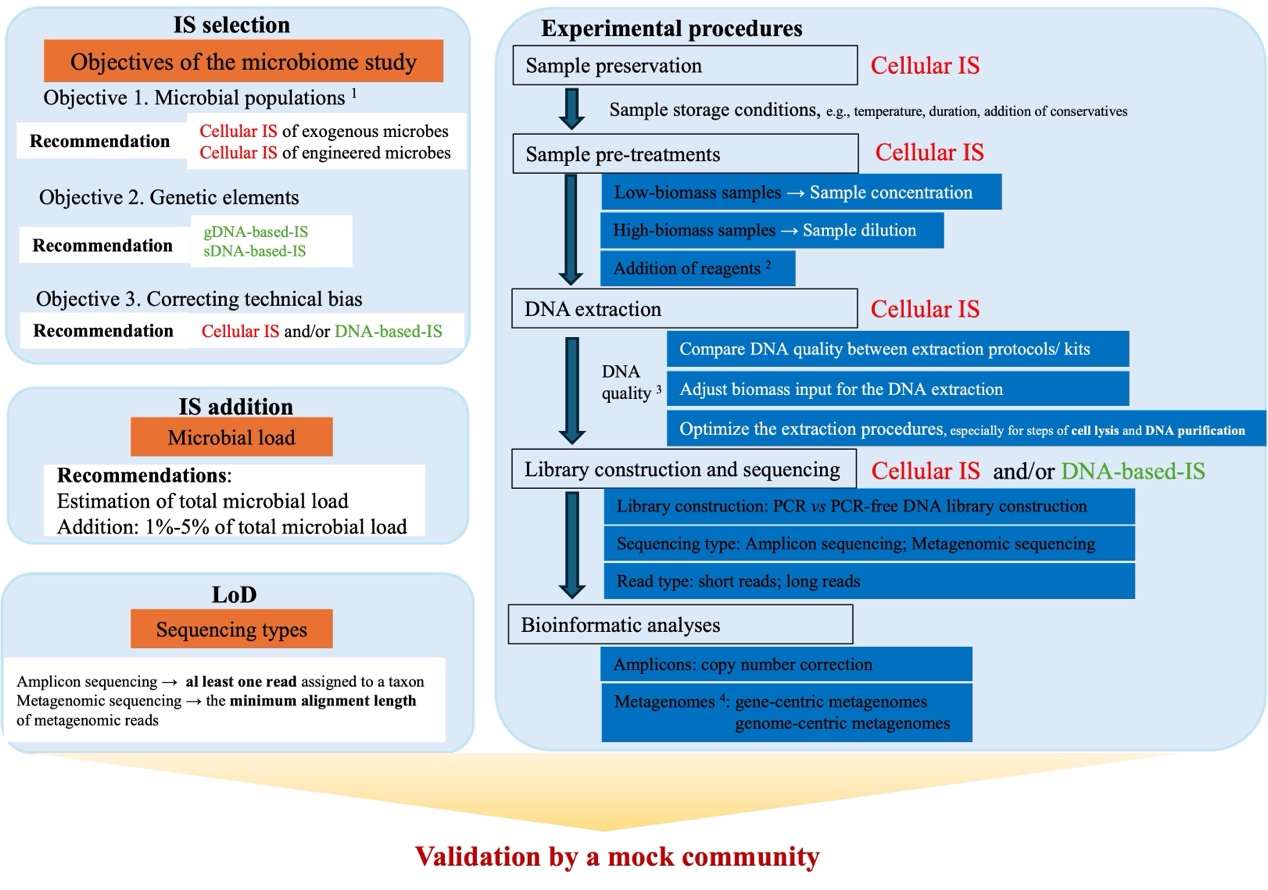
Figure S1.** The technical checklist for the usage of IS-facilitated AQ study. Superscript numbers are technical recommendations. ^1^ If an exogenous microbe is selected as cellular IS or its DNA is used as DNA-based-IS, pre-screening the signals of cellular IS and DNA from the studied environment is necessary. ^2^ Samples are pre-treated by adding reagents, such as PMA dye addition to block the relic DNA signal from sequencing. ^3^ The DNA quality is crucial for Nanopore sequencing. ^4^ Mapping algorithms and their irrespective parameters should be evaluated using simulated datasets.

**References:**

1. APHA. 9215 Heterotrophic plate count. In: Clesceri LS, Greenberg AE, Eaton AD, editors. Standards Methods for the Examination of Water and Wastewater. Washington, DC, USA: American Public Health Association; 1998.

2. APHA. 9221 C. Estimation of bacterial density. In: Clesceri LS, Greenberg AE, Eaton AD, editors. Standards Methods for the Examination of Water and Wastewater. Washington, DC, USA: American Public Health Association; 1998.

3. IDEXX Laboratories I. Quanti-Tray System. https://www.idexx.com/en/water/water-products-services/quanti-tray-system/, Accessed on March, 24 2024.

4. Gensberger ET, Gössl E-M, Antonielli L, Sessitsch A, Kostić T. Effect of different heterotrophic plate count methods on the estimation of the composition of the culturable microbial community. PeerJ. 2015;3:e862.

5. Van Nevel S, Koetzsch S, Proctor CR, Besmer MD, Prest EI, Vrouwenvelder JS, et al. Flow cytometric bacterial cell counts challenge conventional heterotrophic plate counts for routine microbiological drinking water monitoring. Water Res. 2017;113:191-206.

6. Absher M. Hemocytometer counting. In: Tissue culture. Elsevier; 1973. p. 395-7.

7. Thunyaporn R, Doh I, Lee DW. Multi-volume hemacytometer. Sci Rep. 2021;11(1):14106.

8. APHA. 9216 Direct total microbial count. In: Clesceri LS, Greenberg AE, Eaton AD, editors. Standards Methods for the Examination of Water and Wastewater. Washington, DC, USA: American Public Health Association; 1998.

9. Fry JC. 2 Direct methods and biomass estimation. In: Methods in microbiology. Elsevier; 1990. p. 41-85.

10. Hammes F, Berney M, Wang Y, Vital M, Köster O, Egli T. Flow-cytometric total bacterial cell counts as a descriptive microbiological parameter for drinking water treatment processes. Water Res. 2008;42(1-2):269-77.

11. Prest E, El-Chakhtoura J, Hammes F, Saikaly P, van Loosdrecht MC, Vrouwenvelder JS. Combining flow cytometry and 16S rRNA gene pyrosequencing: a promising approach for drinking water monitoring and characterization. Water Res. 2014;63:179-89.

12. Berney M, Vital M, Hülshoff I, Weilenmann H-U, Egli T, Hammes F. Rapid, cultivation-independent assessment of microbial viability in drinking water. Water Res. 2008;42(14):4010-8.

13. Daims H, Ramsing NB, Schleifer K-H, Wagner M. Cultivation-independent, semiautomatic determination of absolute bacterial cell numbers in environmental samples by fluorescence in situ hybridization. Appl Environ Microbiol. 2001;67(12):5810-8.

14. Foladori P, Bruni L, Tamburini S, Ziglio G. Direct quantification of bacterial biomass in influent, effluent and activated sludge of wastewater treatment plants by using flow cytometry. Water Res. 2010;44(13):3807-18.

15. Props R, Kerckhof F-M, Rubbens P, De Vrieze J, Sanabria EH, Waegeman W, et al. Absolute quantification of microbial taxon abundances. ISME J. 2017;11(2):584-7.

16. Myers JA, Curtis BS, Curtis WR. Improving accuracy of cell and chromophore concentration measurements using optical density. BMC Biophys. 2013;6:1-16.

17. Beal J, Farny NG, Haddock-Angelli T, Selvarajah V, Baldwin GS, Buckley-Taylor R, et al. Robust estimation of bacterial cell count from optical density. Commun Biol. 2020;3(1):512.

18. Solera R, Romero L, Sales D. Determination of the microbial population in thermophilic anaerobic reactor: comparative analysis by different counting methods. Anaerobe. 2001;7(2):79-86.

19. Mei R, Liu W-T. Quantifying the contribution of microbial immigration in engineered water systems. Microbiome. 2019;7(1):1-8.

20. Vignola M, Werner D, Hammes F, King LC, Davenport RJ. Flow-cytometric quantification of microbial cells on sand from water biofilters. Water Res. 2018;143:66-76.

21. Contijoch EJ, Britton GJ, Yang C, Mogno I, Li Z, Ng R, et al. Gut microbiota density influences host physiology and is shaped by host and microbial factors. Elife. 2019;8:e40553.

22. Hammes F, Goldschmidt F, Vital M, Wang Y, Egli T. Measurement and interpretation of microbial adenosine tri-phosphate (ATP) in aquatic environments. Water Res. 2010;44(13):3915-23.

23. Velten S, Hammes F, Boller M, Egli T. Rapid and direct estimation of active biomass on granular activated carbon through adenosine tri-phosphate (ATP) determination. Water Res. 2007;41(9):1973-83.

24. Zhang Z, Qu Y, Li S, Feng K, Wang S, Cai W, et al. Soil bacterial quantification approaches coupling with relative abundances reflecting the changes of taxa. Sci Rep. 2017;7(1):4837.

25. Vance ED, Brookes PC, Jenkinson DS. An extraction method for measuring soil microbial biomass C. Soil Biol Biochem. 1987;19(6):703-7.

26. Jenkinson DS, Powlson DS. The effects of biocidal treatments on metabolism in soil—I. Fumigation with chloroform. Soil Biol Biochem. 1976;8(3):167-77.

27. Brookes P, Landman A, Pruden G, Jenkinson D. Chloroform fumigation and the release of soil nitrogen: a rapid direct extraction method to measure microbial biomass nitrogen in soil. Soil Biol Biochem. 1985;17(6):837-42.

28. Frostegård A, Bååth E. The use of phospholipid fatty acid analysis to estimate bacterial and fungal biomass in soil. Biology and Fertility of soils. 1996;22:59-65.

29. Borchardt MA, Boehm AB, Salit M, Spencer SK, Wigginton KR, Noble RT. The environmental microbiology minimum information (EMMI) guidelines: qPCR and dPCR quality and reporting for environmental microbiology. Environ Sci Technol. 2021;55(15):10210-23.

30. Pabinger S, Rödiger S, Kriegner A, Vierlinger K, Weinhäusel A. A survey of tools for the analysis of quantitative PCR (qPCR) data. Biomol Detect Quantif. 2014;1(1):23-33.

31. Pfaffl MW. Quantification strategies in real-time PCR. AZ of quantitative PCR. 2004;1:89-113.

32. Barlow JT, Bogatyrev SR, Ismagilov RF. A quantitative sequencing framework for absolute abundance measurements of mucosal and lumenal microbial communities. Nat Commun. 2020;11(1):2590.

33. Dutton CM, Christine P, Sommer SS. General method for amplifying regions of very high G+ C content. Nucleic Acids Res. 1993;21(12):2953-4.

34. Suzuki MT, Giovannoni SJ. Bias caused by template annealing in the amplification of mixtures of 16S rRNA genes by PCR. Appl Environ Microbiol. 1996;62(2):625-30.

35. Soergel DA, Dey N, Knight R, Brenner SE. Selection of primers for optimal taxonomic classification of environmental 16S rRNA gene sequences. ISME J. 2012;6(7):1440-4.

36. Walker AW, Martin JC, Scott P, Parkhill J, Flint HJ, Scott KP. 16S rRNA gene-based profiling of the human infant gut microbiota is strongly influenced by sample processing and PCR primer choice. Microbiome. 2015;3(1):1-11.

37. Hindson BJ, Ness KD, Masquelier DA, Belgrader P, Heredia NJ, Makarewicz AJ, et al. High-throughput droplet digital PCR system for absolute quantitation of DNA copy number. Anal Chem. 2011;83(22):8604-10.

38. Ding J, Xu X, Deng Y, Zheng X, Zhang T. Comparison of RT-ddPCR and RT-qPCR platforms for SARS-CoV-2 detection: Implications for future outbreaks of infectious diseases. Environment International. 2024;183:108438.

39. Li F, Liu J, Maldonado-Gómez MX, Frese SA, Gänzle MG, Walter J. Highly accurate and sensitive absolute quantification of bacterial strains in human fecal samples. Microbiome. 2024;12(1):168.

40. Tan LL, Loganathan N, Agarwalla S, Yang C, Yuan W, Zeng J, et al. Current commercial dPCR platforms: Technology and market review. Crit Rev Biotechnol. 2023;43(3):433-64.

41. Pinheiro LB, Coleman VA, Hindson CM, Herrmann J, Hindson BJ, Bhat S, et al. Evaluation of a droplet digital polymerase chain reaction format for DNA copy number quantification. Anal Chem. 2012;84(2):1003-11.

42. Stämmler F, Gläsner J, Hiergeist A, Holler E, Weber D, Oefner PJ, et al. Adjusting microbiome profiles for differences in microbial load by spike-in bacteria. Microbiome. 2016;4:1-13.

43. Ji BW, Sheth RU, Dixit PD, Huang Y, Kaufman A, Wang HH, et al. Quantifying spatiotemporal variability and noise in absolute microbiota abundances using replicate sampling. Nature methods. 2019;16(8):731-6.

44. Camacho-Sanchez M. A new spike-in-based method for quantitative metabarcoding of soil fungi and bacteria. Int Microbiol. 2024; 27(3):719-730.

45. Yang Y, Che Y, Liu L, Wang C, Yin X, Deng Y, et al. Rapid absolute quantification of pathogens and ARGs by nanopore sequencing. Sci Total Environ. 2021:152190.

46. Wang C, Yang Y, Wang Y, Wang D, Xu X, Wang Y, et al. Absolute quantification and genome-centric analyses elucidate the dynamics of microbial populations in anaerobic digesters. Water Res. 2022;224:119049.

47. Yang Y, Deng Y, Shi X, Liu L, Yin X, Zhao W, et al. QMRA of beach water by Nanopore sequencing-based viability-metagenomics absolute quantification. Water Res. 2023:119858.

48. Smets W, Leff JW, Bradford MA, McCulley RL, Lebeer S, Fierer N. A method for simultaneous measurement of soil bacterial abundances and community composition via 16S rRNA gene sequencing. Soil Biol Biochem. 2016;96:145-51.

49. Venkataraman A, Parlov M, Hu P, Schnell D, Wei X, Tiesman JP. Spike-in genomic DNA for validating performance of metagenomics workflows. BioTechniques. 2018;65(6):315-21.

50. Lin Y, Gifford S, Ducklow H, Schofield O, Cassar N. Towards quantitative microbiome community profiling using internal standards. Appl Environ Microbiol. 2019;85(5):e02634-18.

51. Crossette E, Gumm J, Langenfeld K, Raskin L, Duhaime M, Wigginton K. Metagenomic quantification of genes with internal standards. MBio. 2021;12(1):10.1128/mbio. 03173-20.

52. Hardwick SA, Chen WY, Wong T, Kanakamedala BS, Deveson IW, Ongley SE, et al. Synthetic microbe communities provide internal reference standards for metagenome sequencing and analysis. Nat Commun. 2018;9(1):3096.

53. Tourlousse DM, Yoshiike S, Ohashi A, Matsukura S, Noda N, Sekiguchi Y. Synthetic spike-in standards for high-throughput 16S rRNA gene amplicon sequencing. Nucleic Acids Res. 2017;45(4):e23-e.

54. Tkacz A, Hortala M, Poole PS. Absolute quantitation of microbiota abundance in environmental samples. Microbiome. 2018;6(1):110.

55. Zemb O, Achard CS, Hamelin J, De Almeida ML, Gabinaud B, Cauquil L, et al. Absolute quantitation of microbes using 16S rRNA gene metabarcoding: A rapid normalization of relative abundances by quantitative PCR targeting a 16S rRNA gene spike‐in standard. MicrobiologyOpen. 2020;9(3):e977.

56. Wang S, Wu Q, Han Y, Du R, Wang X, Nie Y, et al. Gradient internal standard method for absolute quantification of microbial amplicon sequencing data. Msystems. 2021;6(1):10.1128/msystems. 00964-20.

57. Jiang S-Q, Yu Y-N, Gao R-W, Wang H, Zhang J, Li R, et al. High-throughput absolute quantification sequencing reveals the effect of different fertilizer applications on bacterial community in a tomato cultivated coastal saline soil. Science of the Total Environment. 2019;687:601-9.
